# Changes in the Circulation of Common Respiratory Pathogens among Hospitalized Patients with Influenza-like Illnesses in the Lazio Region (Italy) during Fall Season of the Past Three Years

**DOI:** 10.3390/ijerph19105962

**Published:** 2022-05-13

**Authors:** Giuseppe Sberna, Eleonora Lalle, Maria Beatrice Valli, Licia Bordi, Anna Rosa Garbuglia, Alessandra Amendola

**Affiliations:** Laboratory of Virology, National Institute for Infectious Diseases “Lazzaro Spallanzani” IRCCS, Via Portuense 292, 00149 Rome, Italy; giuseppe.sberna@inmi.it (G.S.); eleonora.lalle@inmi.it (E.L.); mariabeatrice.valli@inmi.it (M.B.V.); licia.bordi@inmi.it (L.B.); annarosa.garbuglia@inmi.it (A.R.G.)

**Keywords:** COVID-19, SARS-CoV-2, respiratory pathogens, respiratory syncytial virus, rhinovirus

## Abstract

A descriptive analysis of common respiratory pathogens (CRPs) detected in nasopharyngeal swabs (NPSs) from hospitalized patients with influenza-like illness during the fall seasons of the past three years, 2019–2021, in the Lazio region, Italy, was conducted to assess whether or not CRP circulation changed because of COVID-19 during the fall season. The results observed in a total of 633 NPSs subjected to molecular diagnosis for CRPs by multiplex PCR assay during the autumn seasons (exactly from week 41 to week 50) were compared with each other. In 2019, in 144 NPSs, the more represented CRPs were rhinovirus/enterovirus (7.6%) and influenza A/B (4.2%). In 2020, 55 (21.6%) out of 255 NPSs resulted positive for SARS-CoV-2 and, except for one case of *Legionella pneumophila*, the CRPs detected were exclusively rhinovirus/enterovirus (4.7%). In 2021, among 234 NPSs, 25.6% resulted positive for SARS-CoV-2, 14.5% for respiratory syncytial virus (RSV), and 12.8% for rhinovirus/enterovirus. Compared with 2019, in 2020, CRP circulation was severely limited to a few cases; in 2021, instead, infections by RSV (detected also among adults), rhinovirus/enterovirus, and other respiratory pathogens were observed again, while influenza was practically absent. The comparison of the CRPs detected in the NPSs depicts a different circulation in the Lazio region during the last three fall seasons. CRP monitoring has a direct impact on the prevention and control strategies of respiratory infectious diseases, such as the non-pharmacological interventions implemented in response to the COVID-19 pandemic. Future studies should investigate the impact of specific interventions on the spread of respiratory infections.

## 1. Introduction

The global concern caused by coronavirus disease 2019 (COVID-19) has generated worldwide strict non-pharmacological interventions (NPIs), that is, social restrictions including mandatory face mask wearing in public, physical distancing, case isolation, contact tracing, quarantine for contacts, and general lockdown. Although NPIs have been applied differently in Europe and over time, and, also, in Italy a series of governmental measures [1] regulated these rules according to the local trend of the pandemic, the profound changes induced in behavioral habits of the population modified the common epidemiology of respiratory infections [2,3].

In Italy, although the first SARS-CoV-2 infections were diagnosed at the National Institute for Infectious Diseases “L. Spallanzani” IRCCS, in Rome, Lazio region, at the end of January 2020 [4], the COVID-19 epidemic has spread since the end of February 2020. A previous paper from our group demonstrated that, from February to April 2020, the spread of the SARS-CoV-2 epidemic did not affect the seasonal trends of other common respiratory pathogens (CRPs) in the Lazio region, as it took over when the circulation of these viruses was already underway among the population [5]. Other studies describing the epidemiological situation of CRPs in Northern Italy in these first months of 2020 depicted similar trends among hospitalized adult patients and children [6,7].

During the fall season of 2020, the second pandemic wave throughout Europe imposed again severe NPIs, such as the closure of schools and workplaces, contact tracing, and quarantine. These measures contributed to containing CRP circulation because their spread occurs mainly by contact and droplet routes, and numerous papers described this new shaping of the epidemic curve [2,3,7,8].

In 2021, Italy was facing the fourth wave of the COVID-19 epidemic [9], though in presence of minor NPIs (for example, face masks in public open places were no longer mandatory) thanks to the 85.4% of Italian people with full COVID-19 vaccinations [10]. In this period, the number of influenza viruses isolated from reference laboratories was very low (only 2% of the total swabs analyzed) [9]. By contrast, an increase in cases with flu-like respiratory syndromes not related to SARS-CoV-2 infection was observed in Northern Italy and in the Lazio region, especially due to RSV and rhinovirus [6,7,8,9].

Seasonality patterns of CRPs are well established and depend on geographic area and climate (i.e., influenza and RSV show a peak in winter months; rhinovirus circulates year round with maximum of incidence in spring and fall, while all other respiratory viruses circulate throughout the entire year) and on their epidemiology: influenza, rhinovirus, RSV, human coronaviruses, and human metapneumovirus cause significant morbidity and mortality especially in older adults and those with underlying comorbidities; in children, instead, the pathogens most frequently observed include rhinovirus, RSV, influenza, parainfluenza, and adenovirus. However, social behavior could also impact on the circulation of these pathogens [2,3,11]. In the last three years, the social context and the possibility of the CRPs to circulate among the population were quite different; in fact, differently from 2019, in the following two years unprecedented social restrictive measures and massive SARS-CoV-2 and flu vaccination have been introduced.

The aim of this study was to verify if the seasonal circulation of CRPs (no SARS-CoV-2) was different during the fall of the last three years in the Lazio region as a consequence of NPIs adopted during the COVID-19 pandemic. For this purpose, we analyzed data of NPSs obtained from hospitalized patients with respiratory syndromes subjected to molecular diagnosis for CRPs (adenovirus, coronavirus 229E, coronavirus HKU1, coronavirus NL63, coronavirus OC43, Middle East respiratory syndrome, human metapneumovirus, human rhino/entero, influenza A, influenza B, parainfluenza virus 1, parainfluenza virus 2, parainfluenza virus 3, parainfluenza virus 4, respiratory syncytial virus, *Bordetella parapertussis*, *Bordetella pertussis*, *Chlamydia pneumonia*, *Legionella pneumophila*, *Mycoplasma pneumonia*) upon medical request. In particular, the data here described refers to NPSs collected from week 41 to week 50 (from October to December) of the last three years, 2019, 2020, and 2021. Unlike most similar published articles, this study analyzed the circulation of CRP in a special group of patients, those hospitalized, which may represent a sentinel indicator of the spread of infectious diseases among the population. In addition, we focused on the central weeks of the fall season, because it is a time when respiratory pathogens that usually will give rise to following epidemic peaks begin to circulate.

## 2. Materials and Methods

**Clinical samples.** Data analyzed in this study came from samples (NPSs) belonging to patients hospitalized for acute respiratory syndrome at the main academic hospitals and private clinics of Rome, in the Lazio region, Italy. The samples were sent to the Laboratory of Virology of the National Institute for Infectious Diseases L. Spallanzani during the fall season (from week 41 to week 50) of the years 2019, 2020, and 2021 upon medical request for the molecular diagnosis of respiratory pathogens. A total of 633 nasopharyngeal swabs (NPSs) were evaluated.

**Laboratory diagnosis.** NPSs were subjected to multiplex PCR-based assay for CRPs immediately after delivery to the diagnostic laboratory by using the commercial kit BIOFIRE^®^ Respiratory Panel 2.1 plus (BioFire Diagnostics LLC, Salt Lake, UT, USA) according to the manufacturer instructions. A volume of 300 μL of fresh NPSs was loaded onto the cartridge supplied by the kit. The BIOFIRE^®^ Respiratory Panel 2.1 plus tested for 23 pathogens (19 viruses, including SARS-CoV-2, and four bacteria) responsible for the most frequent respiratory tract infections, and returns the results in 45 min. The diagnostic system did not allow for the distinguishing between rhinoviruses and other species, whereby, when detected, they are reported as rhino/entero. The assay performed a qualitative assessment of pathogens and did not provide information on the threshold cycle with which pathogens were detected.

**Statistical analysis.** Data management and analyses were performed using GraphPad Prism version 8.00 (GraphPad Software, La Jolla, CA, USA). The comparison of proportions was calculated by Z test with normal approximation assumptions of *p* < 0.05 (MedCalc Software Ltd, Acacialaan, Ostend, Belgium). Due to the small number of available data, circulation of CRPs was described biweekly to better appreciate fluctuations in infections with the progression of the season and among years.

**Ethical Issue.** The study was conducted in accordance with the Declaration of Helsinki and with the protocol code no. 70, approved on 17 December 2018, by the Institutional Review Board of the National Institute for Infectious Diseases L. Spallanzani, IRCCS, according to which the study protocol described here did not provide for the signing of an informed consent by the patients since no further samples were taken other than those performed for diagnostic purposes. The data of biological samples collected for diagnostic purposes were used only after their complete anonymization and tests results had no impact on the clinical management of patients. Furthermore, the analysis of genetic data was not conducted.

## 3. Results

During the three periods analyzed (weeks 41–50 of 2019, 2020, and 2021), 633 NPSs were tested for CRPs. The NPSs used in this study were sent to the laboratory for the molecular diagnostic identification of non-SARS-CoV-2 respiratory pathogens. As the diagnostic system used here allowed for the recognizing SARS-CoV-2, the novel coronavirus was detected in numerous samples, as expected, and reported here; however, it did not influence the calculated percentage of positivity for CRPs, as the molecular test was performed only for the search of CRPs (no SARS-CoV-2). Among the samples of 2019, children samples were not included in our analysis for lack of samples. During 2020, a higher number of samples derived from male patients. A brief description of demographic characteristics of the cohort of NPS donors is shown in Table 1.

In the fall season of 2020 and 2021, different NPIs were active, as shown in Table 2. In fact, while in 2020 measures were aimed to prevent the circulation of SARS-CoV-2 through the cancellation of mass gatherings, closure of schools, and the stopping of cultural and business meetings, limiting peoples’ mobility, in 2021, the introduction of mass vaccination in the population and the institution of the green pass allowed the reduction of NPIs, favoring the recovery of people mobility (Table 2).

During the fall season of 2019, when SARS-CoV-2 was not yet present in Italy [1], the respiratory pathogens detected among 144 NPSs were: 11 (7.6%) rhino/entero, 6 (4.2%) influenza A/B, 3 (2.1%) *Mycoplasma pneumonia*, 3 (2.1%) parainfluenza 1–4, 1 (0.7%) RSV, and 1 (0.7%) *Legionella pneumophila* (Table 3).

In the period of 2020, Italy was facing the second wave of SARS-CoV-2 infection [12]. Among the 255 NPSs analyzed, 55 (17.64%) were SARS-CoV-2 positive and 13 (5.09%) resulted positive for CRPs (Table 3). Except for one (0.4%) sporadic case of *Legionella pneumophila*, the other respiratory pathogens detected in 12 specimens were (4.7%) rhino/entero (Table 3).

A different scenario appears in 2021; analyzing 234 NPSs, besides 60 samples (25.6%) positive for SARS-CoV-2, 95 (40.59%) NPSs were positive for CRPs (Table 3), indicating that CRPs started to circulate again in 2021 as compared with 2020. In more detail, 34 (14.5%) NPSs resulted positive for RSV, 30 (12.8%) for rhino/entero, 13 (5.6%) for metapneumovirus, 5 (2.1%) positive samples for influenza A/B, 5 (2.1%) for parainfluenza 1–4, 5 (2.1%) for coronavirus 229E, 2 (0.9%) for coronavirus OC43, and 1 (0.4%) for adenovirus (Table 3).

**Figure 1:**
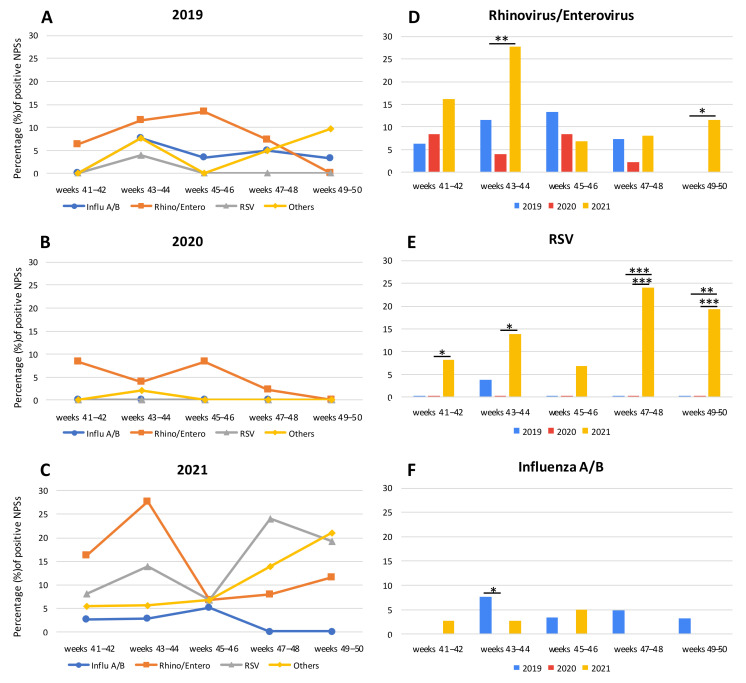
**Biweekly percentage of NPSs positive for CRPs during the fall season of 2019, 2020, and 2021.** (**A**–**C**): Seasonal circulation of CRP-positive samples during fall 2019, 2020, and 2021, respectively. (**D**–**F**): Biweekly of CRP-positive samples in comparison with the same interval periods during 2019, 2020, and 2021. Asterisks indicate statistically significant differences determined by Student’s *t* test (*** = *p* < 0.001; ** = *p* < 0.01; * = *p* < 0.05; no asterisk = not significant).

CRP-positive samples during the fall of 2019, 2020, and 2021 are represented in Figure 1A–C, respectively, and, to better appreciate the seasonal circulation of CRPs, the data were represented by dividing the study periods into 2 weeks intervals. In 2019, the number of CRPs was similar to the previous years [13]. In fact, influenza A/B exhibited a fluctuating trend: 0.0% of frequency in the first two weeks, a peak of 7.7% at 43–44 weeks, a slight flexion at 45–46 weeks (3.3%) remaining stable until the end of the period analyzed (4.9% at 47–48 weeks and 3.2% at 49–50 weeks) (Figure 1A). Rhino/entero showed an increase from 41–42 (6.3%) to 45–46 weeks (13.3%), decreasing at 47–48 weeks (7.3%), and reaching 0.0% in the last two weeks analyzed. RSV had only one peak at 43–44 weeks (3.9%), while in the other weeks it was not present at all (Figure 1A).

In 2020, a drastic decrease of CRPs was observed in our laboratory-based data. However, only rhino/entero remained constantly detected: it was at 41–42 weeks with a frequency of 8.3%, which halved thereafter at 43–44 weeks (3.9%), reaching again the frequency of 8.3% at 45–46 weeks. In the last four weeks it decreased, until reaching 0.0% (Figure 1B).

In 2021, a general CRP rebound was observed. A high percentage of rhino/entero was detected during 41–42 weeks in 16.2% of samples, peaking at 43–44 weeks (27.8%), decreasing at 45–46 weeks (6.8%), and increasing again from 47–48 weeks (8.0) to 49–50 weeks (11.5%) (Figure 1C). In parallel, a high percentage of RSV was observed throughout the period analyzed: it was 8.1% of the positive samples at 41–42 weeks, 13.9% at 43–44 weeks, 6.8% at 45–46 weeks, 24.0% at 47–48 weeks, and 19.2% at 49–50 weeks (Figure 1C). Instead, samples positive for influenza A/B were 2.7% and 2.8% in 41–42 and in 43–44 weeks, respectively; they increased at 45–46 weeks (5.1%) and decreased thereafter until reaching 0.0% at 47–48 weeks (Figure 1C), in agreement with the observed Italian national impact of the previous year [13]. Interestingly, other CRPs were detectable in a large number of NPSs, though not present or rarely detected in the previous two years with a sudden increase in the last two weeks considered (21.2%): i.e., metapneumovirus, parainfluenza 1–4, coronavirus 229E, coronavirus OC43, and adenovirus (Table 2 and Figure 1C).

Furthermore, despite the small number of samples available to us, the biweekly analysis of CRP-positive samples among the same interval periods showed significant differences during 2019, 2020, and 2021. For example, considering 2020 and 2021, rhino/entero was significantly different at 43–44 weeks and at 49–50 weeks [(*p* = 0.0016 and *p* = 0.0120, respectively, Figure 1D)]. Instead, RSV was detected in a significant percentage of samples during the fall season of 2021 in comparison with the previous years. In particular, considering 2019/2021, the differences were statistically significant: *p* = 0.0008 and *p* = 0.0097 for 47–48 weeks and 49–50 weeks, respectively. Considering 2020/2021, at 41–42 weeks *p* = 0.0258, at 43–44 weeks *p* = 0.0064, at 47–48 weeks *p* = 0.0005, and at 49–50 weeks *p* = 0.0009 (Figure 1E). Notably, in 2021, the high number of NPSs which resulted RSV positive was obtained not only from infants (73.5% of the RSV-positive specimens analyzed), but also from adults patients (26.5% of RSV-positive NPSs). Adults with RSV had a median age of 67 years (min-max: 32–99). Considering influenza A/B, a significant difference (*p* = 0.0462) was observed only in 43–44 weeks between 2019 and 2020, but in general it has always been detected in a few samples (Figure 1F).

Interestingly, in 13 (2.1%) NPSs, co-infections were found. In particular, in 2019, one sample showed the contemporary presence of influenza A and parainfluenza 4 and, in 2020, one NPS showed SARS-CoV-2 and rhino/entero. In 2021, on the contrary, samples with the simultaneous presence of two pathogens were more numerous (11): one was positive for influenza A and rhino/entero; one NPS showed parainfluenza 3 and RSV; two NPSs were positive for rhino/entero and RSV; parainfluenza 4 and rhino/entero was detected in one; one sample had SARS-CoV-2 and rhino/entero; one showed influenza A and B; in one SARS-CoV-2 and influenza A were detected; one sample was positive for metapneumovirus and rhino/entero; metapneumovirus was also found in another one specimen co-infected with coronavirus 229E; and one NPS showed RSV and coronavirus 229E.

## 4. Discussion

In this study, a descriptive analysis based exclusively on diagnostic results of NPSs was carried out to assess whether or not the COVID-19 pandemic had an impact on the observed circulation pattern of CRPs among hospitalized patients with acute respiratory syndromes.

Our data showed that, in the Lazio region, CRPs have been circulating in a different manner during the fall season of the last three years. The analysis was conducted biweekly to compare the number of positive samples between one year and another. In 2021, notably, a conspicuous presence of RSV was observed at a level significantly higher compared with the other two years, and an unusual increase in positive specimens was among adult patients. Rhino/entero was also detected in numerous samples in the same period. In 2020, the widespread circulation of SARS-CoV-2 and the phenomenon of viral competition [14,15], in parallel with the NPIs adopted against the COVID-19 pandemic, could have contributed to the massive reduction of the circulation of CRPs, as also observed in other studies [2,3,11]. In fact, our data show an almost absent circulation of CRPs. In the following year, 2021, the extensive SARS-CoV-2 and influenza vaccination coverage achieved in the population could explain the lower rate of SARS-CoV-2 and flu infections compared with that observed in the previous year. However, for the other CRPs, in particular rhino/entero and RSV, a resumption of circulation among the population was observed, despite social distancing restrictions still in effect, probably due to the particular characteristics of these pathogens [2,3].

In Italy, RSV is, in general active, in the period spanning from October/November to March/April, with peak incidence in January/February [16,17]. Historically, RSV was typical in children [16,17] and, as reported in a recent study [18], RSV showed increased detection rates in children during the COVID-19 epidemic [19,20]. In a simulation modelling study in the USA [19], the RSV incidence in children should have been almost double in 2021 in respect to a typical RSV season. Our data about RSV, although based on a small number of samples, would seem to be in agreement with this study [19], and that of Bozzola E. [20], since much more RSV positive NPSs from hospitalized infants in the Lazio region were detected in 2021. In the last few years before the COVID-19 pandemic, RSV infections were increasingly recognized also in older adults, as a cause of severe respiratory disease, with considerable complications (including prolonged hospital stays and high mortality rates) [21,22]. Another recent study confirmed this trend, showing that samples from adults with respiratory tract infections had the highest detection rate for RSV in the fall season from 2016 to 2018 [23]. Again, our data are in line with this course for RSV in Italy, also in the year 2021. Indeed, during the fall season of this year, the presence of RSV was actually detected in a significant proportion of NPSs from adult patients. Even though, in 2020, the severe Italian restrictive measures for SARS-CoV-2 could have contributed to blocking RSV infections, in 2019, in the same seasonal period, the finding of only one positive RSV sample constitutes relative epidemiological evidence of almost no cases of RSV detected in adults in both 2019 and 2020. However, the number of NPSs analyzed in that year was lower compared with that of other years, so the difference observed could reflect this bias. In 2021, the resurgence of RSV seemed to affect the entire European continent, as described by Tin Tin Htar et al. [24] that observed RSV as responsible for a high number of acute respiratory infections and influenza-like illness in adults, although the reasons at the basis of this phenomenon are not yet explained.

For rhinovirus, the circulation among hospitalized patients was constantly observed, also during 2020, despite the reduction in contacts due to the NPIs [25], probably because of its high transmissibility [26] and its ability to bypass the filtering action of face masks [8,27,28]. Moreover, in 2021 we detected rhinovirus in more samples than that observed in the same periods of the previous year. This may be explained not only by the loosening of NPIs, but also by seasonality and seasonal variations (humidity, temperature, and climates) further influencing the circulation of this virus as reported [27].

Although the data presented here refer to a particular group of hospitalized patients living in a part of the territory of the Lazio region, it is important to consider that the increased prevalence of CRPs observed in this particular cohort of people maybe a consequence of SARS-CoV-2 infection. In other words, the CRPs could have returned to circulate due to the greater number of host individuals weakened at the level of the respiratory tract. Such a situation could be the consequence of a previous COVID-19 illness or the consequence of the reduction of immunological defense memory towards these common respiratory pathogens because of a lower exposure to pathogens due to the constant presence of face masks and social distancing. In addition, it is interesting to note that, different from previous years, in the fall of 2021, numerous NPSs showed the simultaneous presence of two respiratory pathogens. The presence of co-infections could be suggestive both of the resumption of CRP circulation and of a general predisposition of the population to respiratory infections as a consequence of the COVID-19 pandemic.

A limit of our study is that it describes CRPs in a restricted and specific segment of the population and, therefore, it could present a picture of respiratory infections slightly different from that of the entire local population composed of healthy individuals with flu-like respiratory syndromes that did not go to the hospital. Furthermore, it was not possible to further expand our analysis as it was carried out on samples received for diagnostic purposes only. In addition, our study did not include NPSs from children among the 2019 samples, so the comparison of the prevalence of RSV and rhino/entero among minors with respect to 2020 and 2021 could be affected. In addition, during the COVID-19 pandemic, the number of NPSs performed was much higher than in the previous year, especially in adults with flu-like symptoms, and this may have influenced the highest number of positive cases for CRPs in 2021.

However, by analyzing samples from hospitalized patients, this report has the advantage of describing the circulation of CRPs among the most vulnerable people, which may represent a sentinel indicator of infectious diseases among the population.

The COVID-19 pandemic has highlighted the effectiveness of NPIs at the level of the entire population in controlling the spread of infectious diseases. However, further large-scale and more in-depth studies should be carried out to evaluate the efficacy of NPIs in the prevention and control strategies of respiratory infectious diseases.

## 5. Conclusions

The results presented here show different circulations of respiratory pathogens during the fall period of the last three years, 2019, 2020, and 2021, in the Lazio region. Differently from fall 2019, a substantial decrease in CRP circulation occurred in 2020 during the second wave of SARS-CoV-2 infection. This scenario was expected, as the Italian SARS-CoV-2 restrictive measures imposed by the pandemic were more severe among the population, with the use of face masks, social distancing, and lockdown. In 2021’s fall, an overall resumption of CRP circulation was observed and, among the CRPs detected, RSV and rhino/entero were ascertained in a considerable number of the samples, not only among infants, but also among adults.

Considering this and other studies describing the current trend in the circulation of CRPs, epidemiological and clinically relevant models are emerging, linked to specific CRPs, which deserve an in-depth evaluation that can help set strategies to control the spread of respiratory infections among the population. In fact, a possible resurgence of the other CRPs, as a consequence of the loosening of the NPIs, especially during the spring and summer seasons, could occur without following the usual well-known seasonal trends.

## Figures and Tables

**Table 1:** Brief description of the NPS samples analyzed.

Year	N. of Analyzed NPSs	N. of Negative Samples (%)	N. of Positive Samples (%)	Male (%)	Female (%)	Median Age (Min–Max)
2019	144	120 (83.3)	24 (16.7)	84 (58.3)	60 (41.7)	59 (17–89)
2020	255	188 (73.7)	67 (26.3)	175 (68.6)	80 (31.4)	61 (5–93)
2021	234	90 (38.5)	144 (61.5)	140 (59.8)	94 (40.2)	59 (0–99)

**Table 2:** Governmental NPIs during fall season of 2019, 2020, and 2021.

	2019	2020	2021
Face mask	No Governmental NPIs (SARS-CoV-2 not present in Italy)	Mandatory indoors and outdoors	Mandatory only indoors
Early closure at 6 pm of restaurants, bars, and other activities		Yes	None
Closures: gyms, swimming pools, spas, and other places of leisure			
Openings: museums only			
All gatherings are prohibited, therefore, also parties, conferences, and fairs			
Sports competitions suspended, except professional ones at national level			
Journeys limited only to necessary needs for work, study, and health			
Curfew between 12 pm and 5 am			
Distance learning and smart working			Yes
COVID-19 vaccination		Not yet available	85.4% of adults vaccinated
Green pass		Not yet available	Mandatory

**Table 3:** List of CRPs detected in positive NPSs in fall of the last three years.

	2019	2020	2021
Influenza A/B	6	-	5
Rhino/Entero	11	12	30
RSV	1	-	34
Parainfluenza 1–4	3	-	5
*Mycoplasma pneumoniae*	3	-	-
*Legionella pneumophila*	1	1	-
Coronavirus OC43	-	-	2
Coronavirus 229E	-	-	5
Metapneumovirus	-	-	13
Adenovirus	-	-	1
SARS-CoV-2	-	55	60
Total	144	255	234

## Data Availability

The data used and/or analyzed during the study are available from the corresponding author on reasonable request.

## Abbreviations

Common respiratory pathogens (CRPs); naso-pharyngeal swabs (NPSs); respiratory syncytial virus (RSV).
